# A preliminary analysis of replicating the biomechanics of helmet therapy for sagittal craniosynostosis

**DOI:** 10.1007/s00381-022-05792-1

**Published:** 2022-12-24

**Authors:** Connor Cross, Hans Delye, Roman H. Khonsari, Mehran Moazen

**Affiliations:** 1 Department of Mechanical Engineering, University College London, London, UK; 2 Department of Neurosurgery, Radboudumc Nijmegen, Nijmegen, The Netherlands; 3 Department of Maxillofacial Surgery and Plastic Surgery, Necker –Enfants Malades Hospital, Assistance Publique – Hôpitaux de Paris, Paris, France

**Keywords:** Calvarial growth, Craniofacial system, Bone formation, Skull

## Abstract

**Purpose:**

The aim of this study was to investigate the biomechanics of endoscopically assisted strip craniectomy treatment for the management of sagittal craniosynostosis while undergoing three different durations of postoperative helmet therapy using a computational approach.

**Methods:**

A previously developed 3D model of a 4-month-old sagittal craniosynostosis patient was used. The strip craniectomy incisions were replicated across the segmented parietal bones. Areas across the calvarial were selected and constrained to represent the helmet placement after surgery. Skull growth was modelled and three variations of helmet therapy were investigated, where the timings of helmet removal alternated between 2, 5, and 8 months after surgery.

**Results:**

The predicted outcomes suggest that the prolonging of helmet placement has perhaps a beneficial impact on the postoperative long-term morphology of the skull. No considerable difference was found on the pattern of contact pressure at the interface of growing intracranial volume and the skull between the considered helmeting durations.

**Conclusion:**

Although the validation of these simulations could not be performed, these simulations showed that the duration of helmet therapy after endoscopically assisted strip craniectomy influenced the cephalic index at 36 months. Further studies require to validate these preliminary findings yet this study can lay the foundations for further studies to advance our fundamental understanding of mechanics of helmet therapy.

**Supplementary Information:**

The online version contains supplementary material available at 10.1007/s00381-022-05792-1.

## Introduction

The neonate skull consists of several bony plates, connected by cranial sutures. Infant skull rapidly grows in the first year of life to accommodate the expanding brain [1, 2]. Craniosynostosis is caused by the premature fusion of one or more of the cranial sutures and occurs in approximately 1:2000 live births [2–4]. The most common form, sagittal craniosynostosis, produces compensatory anteroposterior overgrowth and is represented as a distinct “keel-shaped” skull [5]. If left untreated, defects associated with neurodevelopmental and social complications may arise [6, 7].

The treatment of sagittal craniosynostosis (i.e., scaphocephaly) primarily aims to address the morphological abnormality and restore normal growth [8]. One such treatment is endoscopically assisted strip craniectomy (EAC) followed by helmet therapy. The goal of EAC is to remove the fused portion of the suture and attempt to normalise the skull shape as soon as possible, assisted by the patient-specific helmet that is placed a few days after surgery [9]. The helmet therapy then guides the multidirectional driving force of the expanding brain without being constrictive towards the overall growth.

There is a large body of evidence found in the literature that this treatment modality for craniosynostosis achieves good results, both financially and cosmetically [10–12]. However, it remains unclear as to what degree the early re-opening of the suture (i.e., suturectomy) or the postoperative helmet therapy affects the morphological or functional changes across the skull and brain. Furthermore, the duration of helmet therapy varies between craniofacial centers. Ethically, assessing the cosmetic outcomes of alternating helmet durations within a clinical environment would prove impractical.

The finite element (FE) method is a powerful computational tool used to analyse a wide range of engineering problems [13]. Using these detailed FE models, computational algorithms have been used to investigate the management of craniosynostosis. More advanced models have enabled us to accurately simulate the calvarial growth and bone formation under different types of surgical treatment [14–19]. Such models have the capability to investigate the biomechanics of craniosynostosis and to simulate the outcomes of various surgical parameters, such as postoperative helmet therapy.

The aim of this study was to investigate the biomechanics of endoscopically assisted strip craniectomy treatment while undergoing three different durations of postoperative helmet therapy using a generic FE approach. The study here presents a preliminary investigation into replicating the effects and simulating the outcomes of postoperative helmet therapy years after surgery. The long-term goal of this work is to provide the foundation for further in vitro and in silico experimentation.

## Materials and methods

### CT data

A 3D model of a preoperative sagittal craniosynostosis patient at 4 months of age was developed using computed tomography (CT) data obtained from the Hôpital Necker – Enfants Malades Craniofacial Surgery Unit (Centre de Référence Maladies Rares Craniosténoses et Malformations Craniofaciales CRANIOST, Paris, France). The full ethical protocol for undertaking this study was approved by the institutional review board and committee from the Necker – Enfants Malades University Hospital. Informed consent was granted by the patient’s guardian.

### Image processing and surgical technique

**Fig. 1:**
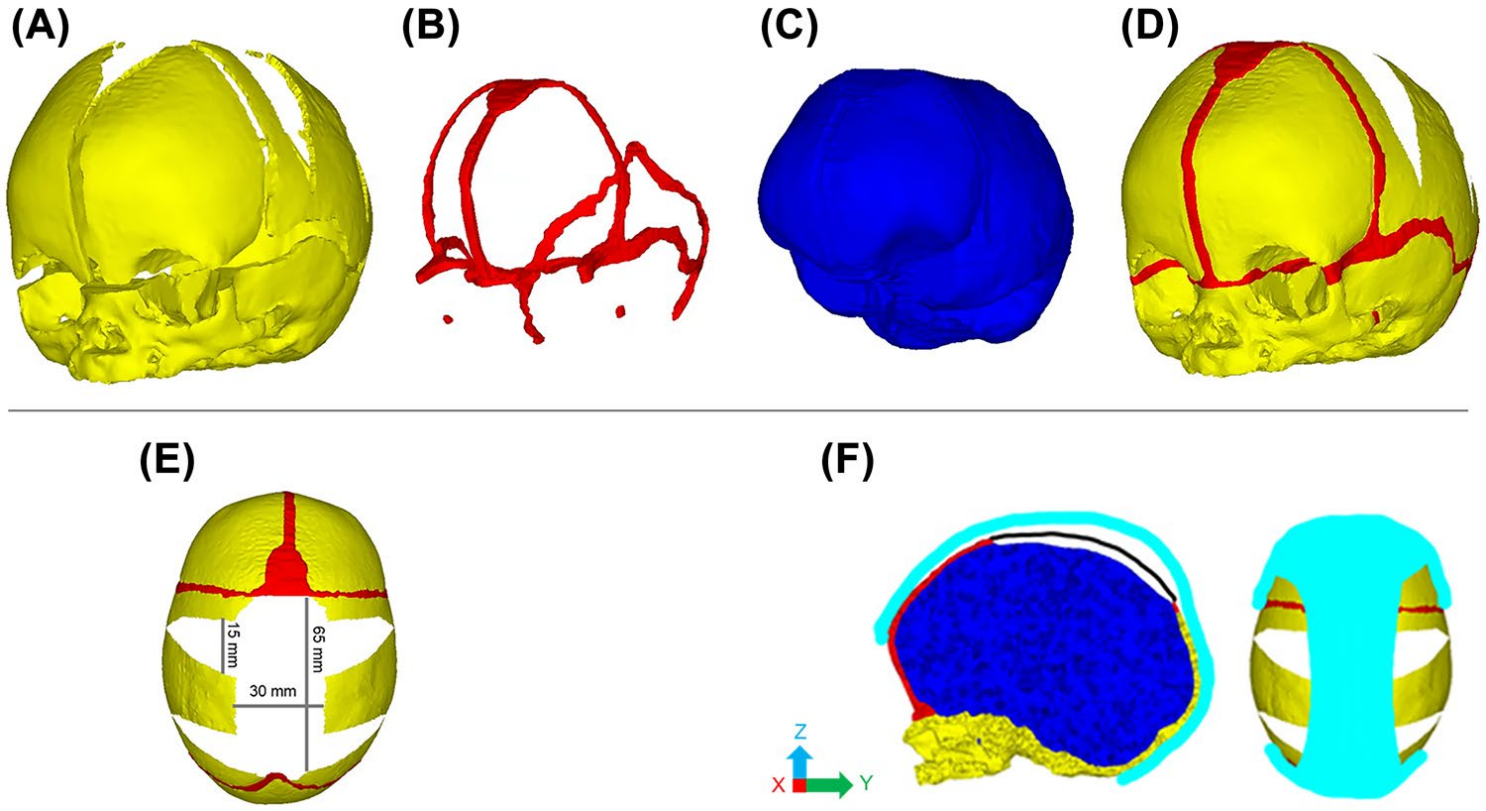
Process of 3D model development. CT imaging was used to segment the calvarial bones (**A**), sutures (**B**), and the ICV (**C**). All were incorporated to create the preoperative model at 4 months of age (**D**), adopted from Cross et al. [17]. The centre-specific craniotomies (marked in white) were replicated across the parietal bone (**E**). Constraints to represent the helmet therapy (light blue) were placed across the temporal, frontal, and parietal bones. While a level of vertex displacement was granted during simulated growth, quantified by a performed sensitivity test (**F**)

Anatomical 3D rendering of the CT data was performed in the imaging processing software, Avizo (Thermo Fisher Scientific, Mass, SA). Segmentation of the calvarial bones, cranial sutures, and the intracranial volume (ICV, i.e., all internal calvarial components) was performed and is displayed in Fig. 1A–D. The calvarial bones were segmented using automatic thresholds to differentiate between the hard and soft tissues. Both the sutures and the ICV were segmented manually.

The EAC technique, as performed at the Radboudumc Centre of Expertise Craniofacial anomalies (Radboudumc Nijmegen, The Netherlands), was replicated across the 3D model under the surgical teams’ guidance and the detailed report of Delye et al. [10]. Figure 1E depicts the replicated craniotomies performed across the 3D model. In short, an anteroposterior suturectomy, measuring a width of approximately 30 mm, was made across the fused sagittal suture to encourage dorsal growth. Four wedge-shaped craniotomies were made across the parietal bones and extended towards the squamosal sutures to promote bitemporal widening. These wedges measured approximately 15 mm wide at their bases.

### Finite element model development

Approximately 4 million quadratic tetrahedral elements were transposed across the complete 3D model in preparation for the finite element analysis that was performed using ANSYS (V19.0; Canonsburg, PA, USA). The program allows for the material properties to be defined as well as the skull growth, bone formation, ICV contact pressure, and helmet therapy to be computationally simulated.

Material properties of the calvarial bones, the cranial sutures, and the ICV were all defined as linear isotropic and assigned an elastic modulus of 421 MPa, 30 MPa, and 10 MPa, respectively [16–20]. The replicated craniotomies were assigned an elastic modulus of 0.3 MPa, to represent the natural “gaps” made in situ and minimise the level of resistance on the simulated growth [16]. Both the ICV and craniotomies Poisson’s ratio was selected as 0.1. A Poisson ratio of 0.3 was assigned to the calvarial bone and the cranial sutures.

### Boundary conditions

To represent the skull growth, a previously adopted thermal expansion analogy was introduced across the ICV of the model [21]. This approach was used to simulate the expansive growth of the ICV across five load steps, from the initial preoperative 4 months of age volume (measuring 659 ml) to the approximate target follow up volume seen at 36 months of age (measuring 1240 ml). At each load step, the age of the model was approximated by correlating the predicted volumes against relevant literature data [22]. This, in turn, allowed for the applicable timing of helmet removal to be determined. As the morphology of the skull shape changes, the geometry of the model was updated at each load step to represent the new skull shape. To avoid rigid body displacement, constraints in all degrees of freedom were placed around the foramen magnum and nasal ridge of the model.

The bone formation across the cranial sutures and the craniotomies during the growth was simulated based on a previous study [18]. In short, the rate and the distance of bone formation across the cranial sutures were dictated by the level of strain (generated by the expansion of the ICV) followed by a predetermined radius extending from the adjoining bony borders (determined by the changes in age at each load step). The bone healing across the craniotomies was controlled only by the level of strain, allowing for spontaneous bone formation away from the bony borders to be permitted. Cranial suture and craniotomy elements that met their relevant conditions had their elastic moduli updated at each load step to represent the effects of osteoblast cell behavior [23]. Further, the calvarial bone components’ elastic modulus was also updated to represent the changes in bone malleability with age.

Estimating the level of loads across the intracranial space (here, the ICV) using surface-to-surface contact elements is a previously used approach for observing and quantifying the pressure changes under simulated growth [16–18]. Although highly informative, clinically, such information may not represent the true pressure distribution or absolute values post-surgery. Nonetheless, in the interests of this work, this method was introduced to the EAC technique for examination. In short, the level of pressure across the ICV surface was captured and quantified. Parameters to minimise the interpenetration between these surfaces during growth were previously established and described elsewhere [16–19].

### Helmet therapy

A simplistic approach to model the effects of helmet therapy was developed (Fig. 1F). The helmet was not represented as a physical geometry across the model, instead nodal constraint was applied to model the effect of the helmet. Here, constraints were applied across the anterior (Y-axis), posterior (Y-axis), and lower bitemporal (X-axis) regions restricting the growth in the applied axis. This prevented relative movement throughout the simulations. A permittable 20-mm level of dorsal displacement (Z-axis) was granted during growth. This value was chosen based on the surgeon’s guidance and from a sensitivity study that is described in the appendix (See: Supplementary Fig. S1 and Table S1). The potential impacts regarding the helmets’ thickness and its material properties were not considered here.

The effects of helmet therapy were introduced to the FE model at 4 months of age, along with the replicated EAC surgery, and remained during the simulated growth period until the helmet was ‘removed’ (i.e., deletion of all helmet-related constraints). The timing of helmet removal alternated across three scenarios, at 2 months, 5 months, and 8 months after surgery, respectively. The latter time points reflects the average duration for patients reported by Delye et al. [9]. A control scenario, where only the EAC surgery was replicated across the model (i.e., No helmet introduced) was used as a comparative scenario.

### Simulations and measurements

All scenarios underwent calvarial growth up to the follow up age of 36 months. The predicted pattern of bone formation was captured at each load step during the simulated calvarial growth. The skull length (glabella to opisthocranion), width (left and right euryons), and cephalic index (skull width divided by the skull length and multiplied by a hundred) were quantified during the calvarial growth. The level of contact pressure across the ICV was captured and compared at each load step during the simulated calvarial growth across all considered scenarios.

## Results

**Fig. 2:**
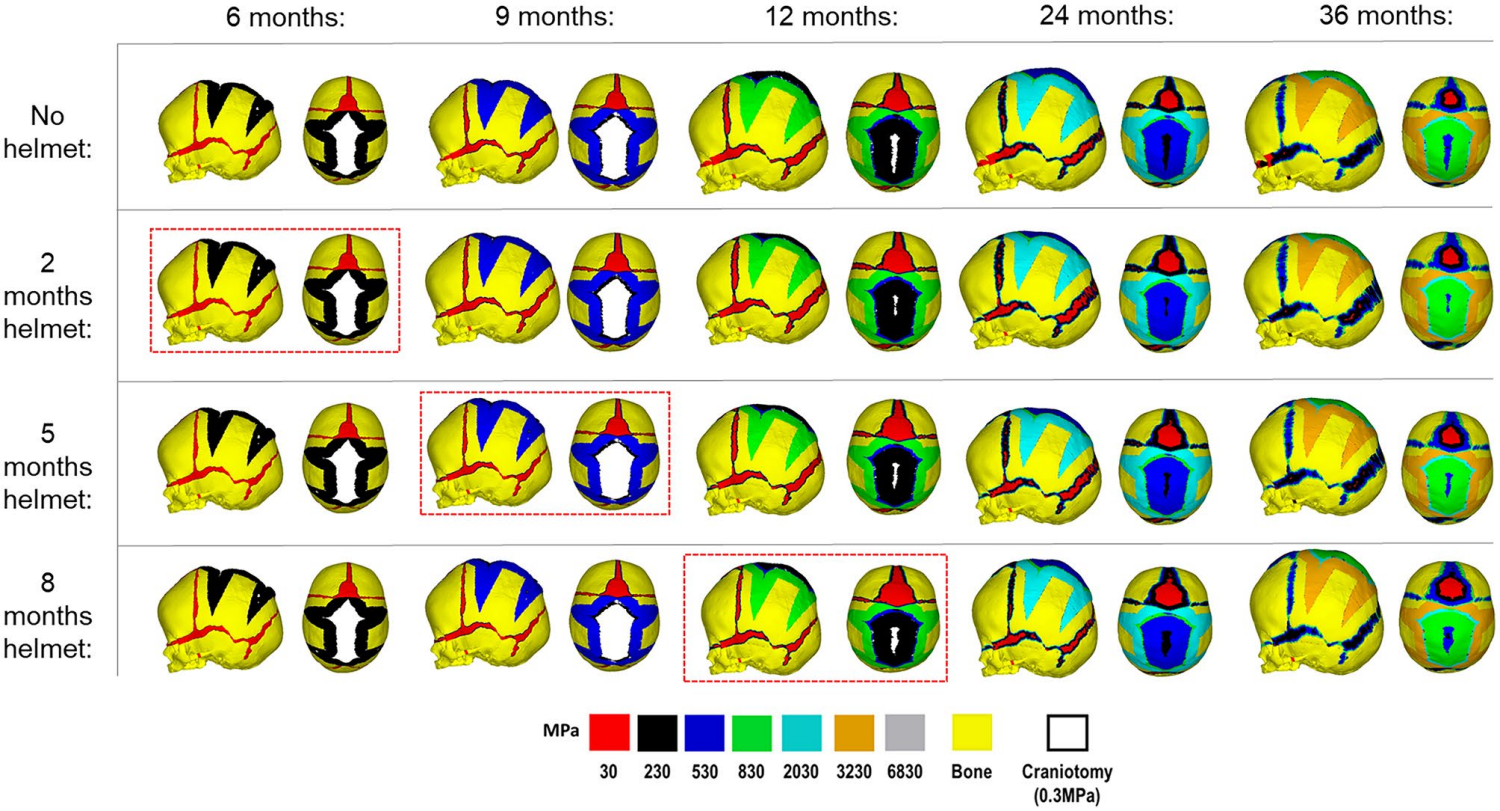
Predicted pattern of bone formation and skull shape with alternating durations of postoperative helmet treatment during the simulated growth. Red dashed boxes indicate the respective time points of helmet removal. Displaying lateral and dorsal views

The predicted patterns of bone formation across the skull and its overall morphology across each helmet scenario are highlighted in Fig. 2. All helmeting scenarios and the single non-helmet scenario achieved craniotomy healing (here, defined as the initial white material no longer being present) by 20 months after surgery. All sutures, disregarding the anterior fontanelle, achieved a similar pattern of bone formation by the final load step of 36 months of age for all scenarios.

While the lack of a postoperative helmet did not impact the level or pattern of bone formation or bone healing during the simulated skull growth, a characteristic dorsal “bulge” was evident by 36 months of age. This was seen to have corrected itself once the helmet had been introduced, regardless of its duration. Prolonging the helmet's removal (i.e., 8 months) was seen to encourage greater bitemporal widening in the long term when compared to the shorter durations (2 and 5 months).

**Fig. 3:**
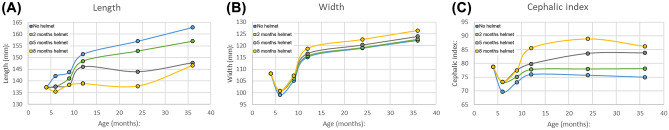
Cephalometric data of the simulated length (**A**), width (**B**), and cephalic index (**C**) from 4 months up to 36 months of age across alternating durations of postoperative helmet treatment

Figure 3 and Table 1 quantify the changes in skull length, skull width and cephalic index across all helmeting scenarios up to the follow up age of 36 months. As each scenario utilised the same preoperative model, all represent identical length (137.2 mm), width (108.1 mm), and cephalic index (78.7) at 4 months of age. By 36 months of age, the greatest length was recorded in the “No helmet” scenario (162.9 mm). The shortest was seen in the 8-month helmeting duration (146.6 mm). A difference of only 4 mm was seen across all simulated scenarios widths by 36 months. The highest was seen in the 8-month helmet duration (126.4 mm) whilst the lowest was in the “No helmet” scenario (122.2 mm). These observations were reflected in the cephalic indexes, where the highest value was achieved by the 8-month duration helmet (86.2), while the lowest was recorded in the “No helmet” scenario (75.0).

**Table 1:** Quantitative data of all cephalometric data for all alternating helmet durations. Entries in italics indicate the respective time points of helmet removal

		6 months	9 months	12 months	24 months	36 months
No helmet	Length (mm)	142.1	143.7	151.4	157.0	162.9
	Width (mm)	99.1	105.1	115.1	118.9	122.2
	Cephalic index	69.7	73.1	76.0	75.7	75.0
2 months helmet	Length (mm)	*137.5*	141.1	148.5	152.8	157.0
	Width (mm)	*100.7*	106.1	115.6	119.2	122.7
	Cephalic index	*73.2*	75.1	77.8	78.0	78.1
5 months helmet	Length (mm)	137.5	*138.3*	146.0	143.9	147.7
	Width (mm)	100.7	*107.2*	116.6	120.3	123.9
	Cephalic index	73.2	*77.5*	79.8	83.6	83.8
8 months helmet	Length (mm)	135.5	138.3	*138.8*	137.8	146.6
	Width (mm)	100.7	107.2	*118.7*	122.6	126.4
	Cephalic index	73.2	77.5	*85.5*	88.9	86.2

**Fig. 4:**
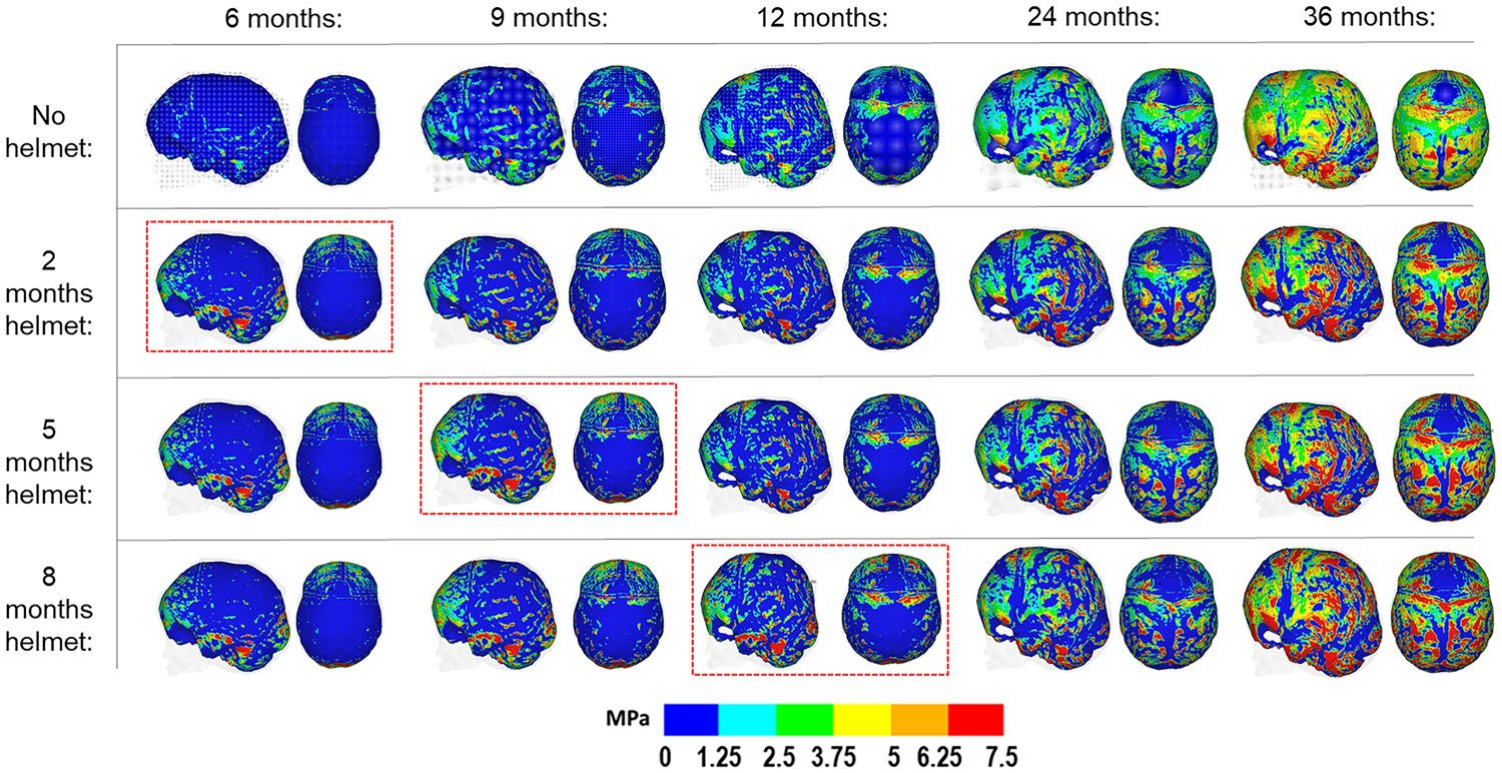
Pattern of contact pressure across the modelled ICV with alternating durations of post-surgical helmet treatment during the simulated growth. Red dashed boxes indicate the respective time points of helmet removal. Displaying lateral and dorsal views. Note that absolute values do not correspond to real intracranial pressure values

Figure 4 highlights the surface ICV contact pressure levels during the simulated growth for all considered scenarios in this study. The initial pressure levels at 4 months of age were not obtained for comparison here (as this was the starting time point of the simulations). Nonetheless, at 36 months of age, largely similar patterns of pressure were captured across all scenarios involving the helmet, regardless of the duration of the placement. Areas of higher pressure were seen across the temporal, occipital, and dorsal regions for all helmet conditions while being slightly lower across the anterior region. The absence of the helmet resulted in a more uniform pattern of pressure across the ICV.

## Discussion

Sagittal craniosynostosis results in over compensatory anteroposterior growth, ventrodorsal shortening, and bitemporal narrowing of the skull. The method of postoperative helmet therapy aims to help guide the skull growth vectors to address these morphological abnormalities after the initial surgery has been performed. Computational models have the potential to optimise the management of this condition by answering key biomechanical-based questions [e.g., 14, 17, 18]. This study assessed the impacts that various durations of postoperative helmets could have on the long-term morphology of the skull.

Due to the lack of standard follow-up CT scans after EAC and the variability of the modelling approaches shown here, mostly regarding the duration of helmeting, the study suffers a lack of morphological or contact pressure validity. On the other hand, the generic FE model used here had been previously validated using patient-specific follow up data which could support a level of validity in this study [16]. The debate on optimising the method of correcting scaphocephaly is still a highly discussed topic within the literature [6, 8]. With the advancements in computational modelling approaches, conclusions to such discussions could be addressed [e.g., 18, 19].

The method of replicating the calvarial growth and bone formation discussed here was adopted from a previous study [18], presenting a promising method of replicating the impacts that postoperative calvarial healing could have on surgical outcomes. However, the modelling approach lacks key biological considerations when compared to the true in vivo conditions [e.g., 1, 23]. In particular, a large level of suture patency was seen 8 months after the replicated surgery. The regenerative abilities of bone during infancy, with rare exceptions, can achieve complete surgical healing weeks after surgery [24]. From a modelling point of view, however, it could be argued that the prolonging of calvarial healing in these simulations allows the model to continue to estimate the long-term postoperative morphology, minimising the constrictions on the growth.

Helmet therapy after EAC has been reported to be a cost-effective method of correction while achieving the overall surgical goals for sagittal craniosynostosis. The technique adopted here is reported by Delye et al. [10], where 10-month postoperative helmeting is the standard practice. Such reports detail overall improvement to the cephalic shape postoperative. Although they are overestimated, such observations were also captured in our simulations. Regarding the discussed method of replicating the helmet in our model, there is a clear distinction between the incorporation of the helmet and the postoperative duration. Most notably is the impact on the length which, unlike the width measurements, showed the overall greatest change. This is an understandable response, due to the greater levels of constraints placed across the frontal and occipital bones vs. the temporal regions (Fig. 1F).

In reality, the average number of helmets produced throughout treatment is two [9], to accommodate for the natural growth of the head. This could grant a level of anteroposterior growth as the newly applied helmet forms the shape of the patient’s skull. However, such a parameter was not considered here. This led to almost zero give in skull lengthening throughout the simulated growth when the helmet was modelled.

An alternative method to the helmet modelling approach shown here, which may address this issue, would be the rendering of a 3D solid helmet model, parameterised to fit and correct the generic FE model used here. However, due to the current computational costs of running these models, this prospect can be considered for future studies.

The study of simulating and comparing the level of contact pressure across the modelling ICV is a relatively new and novel approach for investigating the interaction between growing ICV and the overline calvarial bones across various surgical options [16–18]. Due to the lack of validity in this analysis, the simulations shown here must be interpreted with caution. It is hoped that such simulated results could assist with the interpretation of neurofunctional characteristics years after surgical intervention. Although the correlation between the ICV contact pressure shown here and the defective consequences of functional characteristics is unrealistic, the contact pressure data might be able to give us an indication of the risk of elevated ICP following different surgical strategies [25, 26]. The different scenarios showed no effect of the helmet duration on the pattern of contact pressure on the ICV. Within the literature, the impacts on the morphological outcomes using the helmet therapy approaches have been previously recorded [27, 28]. However, there is limited data which records the cognitive attainments after surgery [29].

## Conclusions

The work presented here provides a novel methodology for simulating the impacts three alternating durations of helmet therapy after EAC have on the skull morphology using the finite element method. Although the validation of these simulations could not be performed, these simulations showed that the duration of helmet therapy after EAC influenced the cephalic index at 36 months, with the highest value achieved by the 8 months duration helmet (86.2), while the lowest was recorded in the “No helmet” scenario (75.0). This study provides critical information which could aid surgeons in understanding the postoperative outcomes of endoscopically assisted strip craniectomy accommodated with postoperative helmet therapy. Further studies aim to replicate the effects of helmet therapy under an in vitro approach.

## Supplementary Information

Below is the link to the electronic supplementary material.

Supplementary file1 (DOCX 1085 KB)

## Data Availability

All data generated or analyzed during this study are included in this article. Further inquiries can be directed to the corresponding author.
