# Supplementary material for: A preliminary analysis of replicating the biomechanics of helmet therapy for sagittal craniosynostosis
Source: Childs Nerv Syst. 2022 Dec 24;39(4):989–96. doi: 10.1007/s00381-022-05792-1 (PMC10160196; doi:10.1007/s00381-022-05792-1)

**Supplementary**

**Table S1:** A sensitivity study investigating the impacts on the morphology when altering the distance between the helmet strip and the modelled skull. The control scenario (i.e., Open gap) replicates the helmet constraints only across the anterior and posterior of the model. The first modelled helmet maintains the anterior and posterior constraints seen in the control, with a 10 mm displacement restriction applied to the dorsal growth of the model (i.e., 10 mm gap) before constraints are applied. The second modelled helmet carries over these parameters with the exception of increasing the allowable dorsal displacement (i.e., 20 mm gap). All scenarios underwent growth of the ICV from 4 months to 12 months of age. Results show the maximum dorsal height, measured from the foramen magnum to the most transverse dorsal point of the model.

**
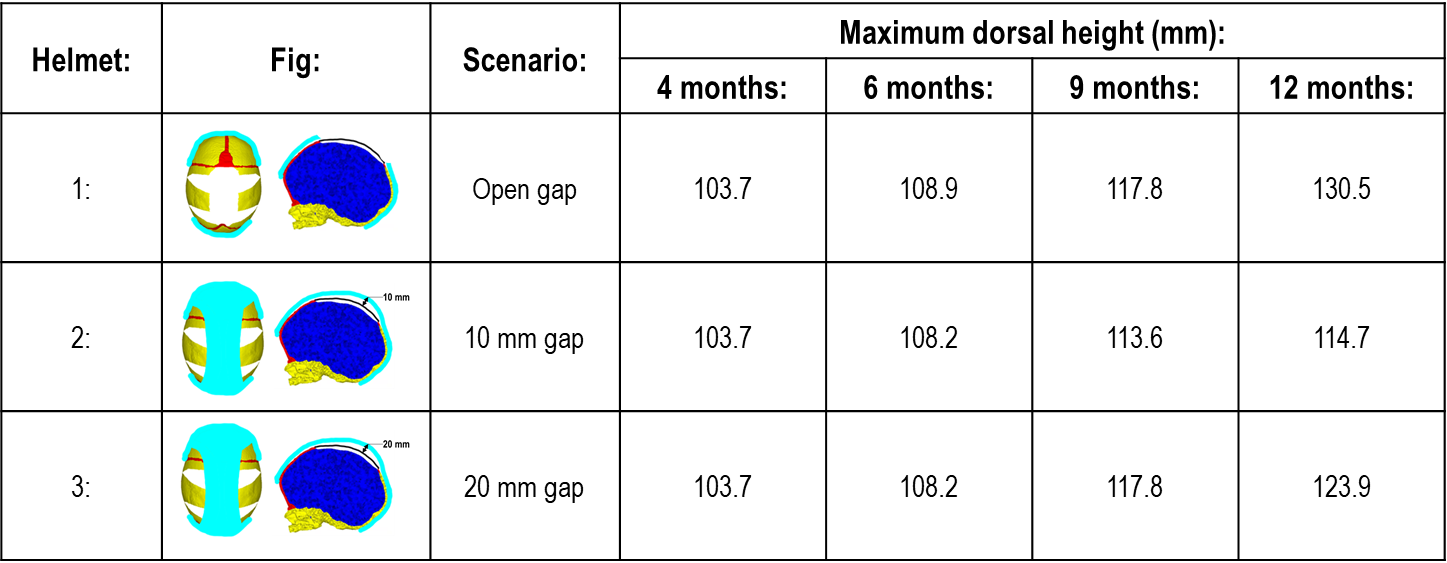
**

**Figure S1**: The simulated pattern of bone formation captured across all sensitivity scenarios. All models presented incomplete craniotomy healing by the 12 months of age mark. A greater level of dorsal bulging (red box) was seen in the ‘open gap’ scenario due to the constraints applied to the anterior and posterior of the skull. Scenario ’10 mm gap’ predicts a severe flattening across the dorsal (black box), accommodated by the greatest lack of calvarial healing. Caused by much of the skull reaching the allowable 10 mm gap threshold. The ‘20 mm gap’ parameter was used throughout all simulations in the main manuscript, as this addressed the dorsal bulging and flattening (blue box) seen in the respective former scenarios.


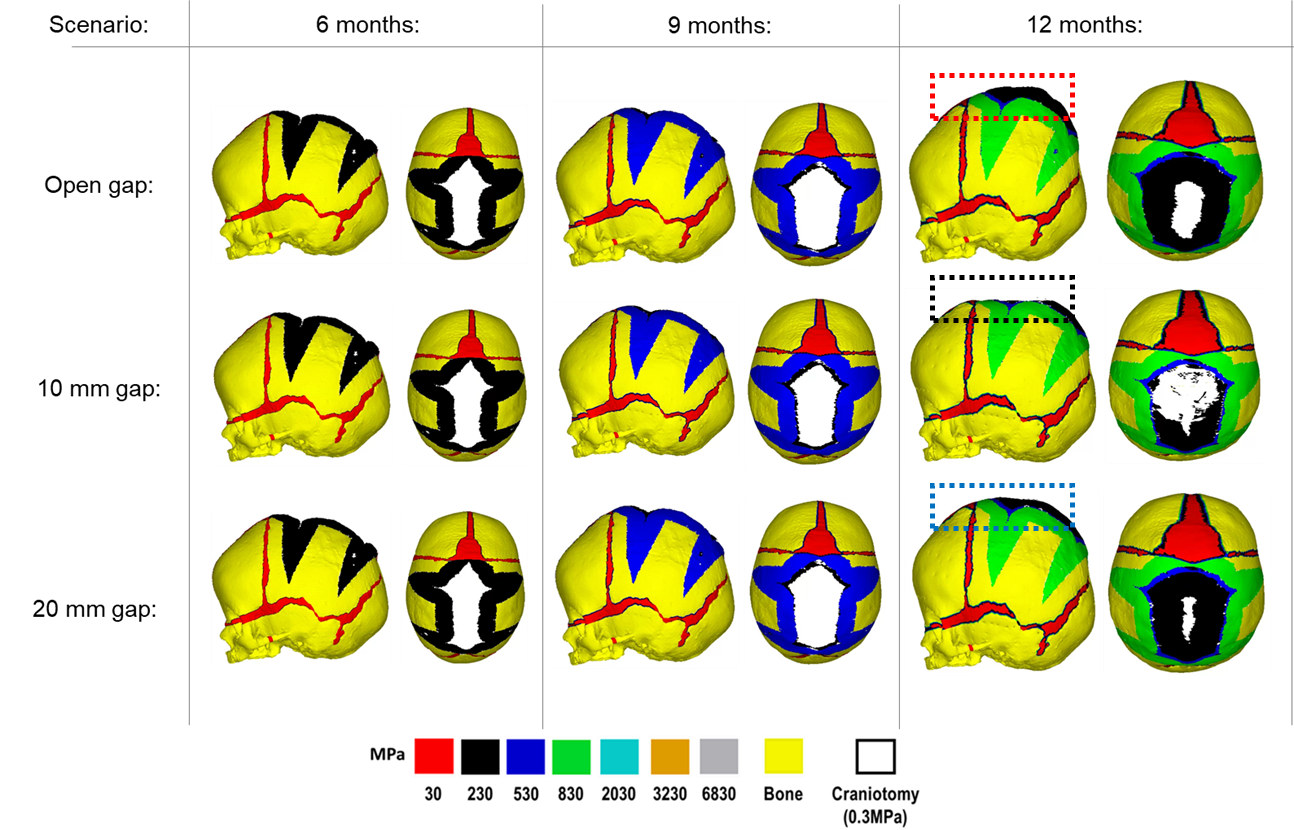

Supplement: Supplementary file 1 — Supplementary file1 (DOCX 1085 KB) [file 381_2022_5792_MOESM1_ESM.docx]
